# Antibacterial Activity of Essential Oils and Their Isolated Constituents against Cariogenic Bacteria: A Systematic Review

**DOI:** 10.3390/molecules20047329

**Published:** 2015-04-22

**Authors:** Irlan Almeida Freires, Carina Denny, Bruna Benso, Severino Matias de Alencar, Pedro Luiz Rosalen

**Affiliations:** 1 Department of Physiological Sciences, Piracicaba Dental School, University of Campinas, Piracicaba, SP 13414-903, Brazil; E-Mails: irlan.almeida@gmail.com (I.A.F.); dennycarina@hotmail.com (C.D.); brunabenso@hotmail.com (B.B.); 2 Department of Agri-food Industry, Food and Nutrition, “Luiz de Queiroz” College of Agriculture, University of São Paulo, Piracicaba, SP 13418-260, Brazil; E-Mail: smalencar@usp.br

**Keywords:** natural products, essential oils, monoterpenes, dental caries, *Streptococcus mutans*, preventive dentistry, clinical trials, isolated compounds

## Abstract

Dental caries remains the most prevalent and costly oral infectious disease worldwide. Several methods have been employed to prevent this biofilm-dependent disease, including the use of essential oils (EOs). In this systematic review, we discuss the antibacterial activity of EOs and their isolated constituents in view of a potential applicability in novel dental formulations. Seven databases were systematically searched for clinical trials, *in situ*, *in vivo* and *in vitro* studies addressing the topic published up to date. Most of the knowledge in the literature is based on *in vitro* studies assessing the effects of EOs on caries-related streptococci (mainly *Streptococcus mutans*) and lactobacilli, and on a limited number of clinical trials. The most promising species with antibacterial potential against cariogenic bacteria are: *Achillea ligustica*, *Baccharis dracunculifolia*, *Croton cajucara*, *Cryptomeria japonica*, *Coriandrum sativum*, *Eugenia caryophyllata*, *Lippia sidoides*, *Ocimum americanum*, and *Rosmarinus officinalis*. In some cases, the major phytochemical compounds determine the biological properties of EOs. Menthol and eugenol were considered outstanding compounds demonstrating an antibacterial potential. Only *L. sidoides* mouthwash (1%) has shown clinical antimicrobial effects against oral pathogens thus far. This review suggests avenues for further non-clinical and clinical studies with the most promising EOs and their isolated constituents bioprospected worldwide.

## 1. Introduction

Despite the advances in public policies so far, dental caries remains the most prevalent and costly oral infectious disease worldwide [1,2], representing a global public health problem to be managed by authorities and dental professionals [2,3]. Effective caries-preventive methods have been developed and amended in the last decades. It is well known that the chemical control of plaque is an effective strategy to prevent dental caries development [4]. The main chemical agents currently available are fluoride [5], chlorhexidine [6], triclosan, cetylpyridinium chloride and natural products [4,7].

In this context, natural products (plant extracts, essential oils and isolated compounds, and marine products) have been proposed as novel therapeutic agents against dental caries [8], in order to minimize the adverse effects of synthetics [9] (e.g., altered taste, mucosal desquamation and tooth staining) as well as to provide effective and safer alternatives for dental caries management. Examples of these natural products include propolis, black and green tea, cacao bean husk, oat hulls, cranberry, and shells of crustaceans, among several others [8].

Essential oils (EOs) have aroused attention among the naturally-occurring bioactive agents with promising antimicrobial activity [10,11]. EOs are a mixture of volatile constituents produced by aromatic plants as secondary metabolites, as a protective mechanism against predators, microorganisms or weather adversities [12,13]. Among the 100,000 known secondary metabolites, EOs account for over 3000, of which about 300 have commercial interest and are used by the food, cosmetic and pharmaceutical industries [10]. The diverse chemical structures of EOs encompass two groups with distinct biosynthetic origins [14]: terpenes (monoterpenes and sesquiterpenes) and terpenoids (isoprenoids), and another group of aliphatic and aromatic compounds (e.g., aldehydes, phenols, among others), all characterized by low molecular weight [12]. Monoterpenes are the major compounds found in EOs [12] and have been found to show potent antibacterial activity against caries-related microorganisms [11,15].

Despite the research progress so far, there have been few studies with EOs approaching their potential application in the field of dentistry. Usually, a few substances from this phytochemical class have been used in anti-plaque and anti-gingivitis mouthwash formulations [16,17,18], hence there is a need for further exploration of EOs with potential use as adjunctive anti-caries chemotherapy.

In this systematic review, we discuss the anti-caries activity of EOs in view of their potential applicability in novel dental formulations. Moreover, the compilation of a vast database from the literature may suggest avenues for further laboratorial and clinical studies with the most promising EOs and their isolated constituents bioprospected worldwide.

## 2. Results

**Figure 1:**
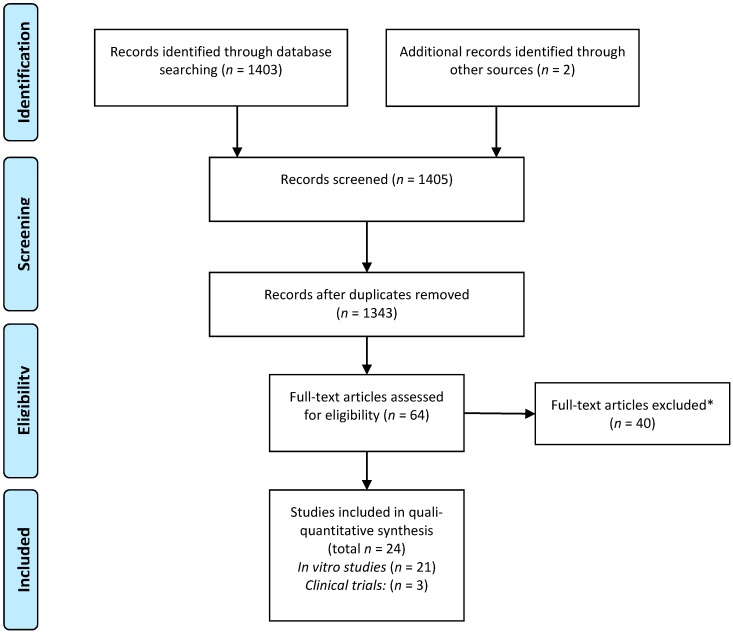
Flow diagram of the search strategy comprising the identification of potentially relevant material, and preliminary screening and final selection of the studies included in this review (based on PRISMA guidelines). * The leading reasons for exclusion of articles were: clinical trials—“score lower than 3 in Jadad’s scale” (see Methods); *in vitro* studies—lack of critical information on chemical profiling, and methodological shortcomings.

According to a previously set strategy, literature searches resulted in 1405 articles, of which 25 met the inclusion criteria and were included in the final review after thorough analysis (Figure 1). A total of 22 *in vitro* studies and three clinical trials addressing the anti-caries properties of EOs and their isolated compounds were selected and will be further discussed herein.

### 2.1. In Vitro Studies

According to the *in vitro* studies analyzed, there was a predominance of tests with planktonic cultures (Table 1, Table 2, Table 3, Table 4, Table 5 and Table 6) rather than mono- or multi-species biofilm cultures (Table 7). Of the 22 studies, 5 (22.72%) tested the effect of the EO on streptococci and lactobacilli biofilms.

#### 2.1.1. Planktonic Studies

##### Crude EOs and Planktonic *S. mutans*

Thirty species were found to have very strong or strong antibacterial activity against *S. mutans*, of which the most promising were *Achillea ligustica* All. (ligurian yarrow) [19], *Cryptomeria japonica* D. Don (sugi) [20], *Croton cajucara* Benth (sacaca) [21], *Baccharis dracunculifolia* DC (broom weed), *Coriandrum sativum* L. (coriander), *Lippia sidoides* Cham. (rosemary-pepper), *Mikania glomerata* Sprengel (guaco) and *Siparuna guianenses* Aubl. (wild lemon) [11], with planktonic MIC values equal to or lower than 100 µg/mL (Table 1).

**Table 1:** *In vitro* antibacterial activity of essential oils against *Streptococcus mutans.*

Plant Species	Source	Microorganism	MIC (µg/mL)	MBC (µg/mL)	Score	Ref.
*Achillea ligustica* All.	Inflorescences	DSM 20523	155	nt	+++	[19]
*Achillea ligustica* All.	Leaves	DSM 20523	155	nt	+++	[19]
*Achillea ligustica* All.	flowering aerial parts	DSM 20523	38	nt	++++	[19]
*Achillea ligustica* All.	Flowers	DSM 20523	155	310	+++	[22]
*Achillea ligustica* All.	vegetative parts	DSM 20523	39	39	++++	[22]
*Ageratum conyzoides*	Leaves	ATCC 25175	4000	nt	−	[23]
*Aloysia gratissima*	Leaves	UA 159	125–250	250–500	+++	[11]
*Aloysia triphylla*	Leaves	UA 159	125–250	125–250	+++	[11]
*Alpinia speciosa*	Root	UA 159	125–250	250–500	+++	[11]
*Artemisia camphorata* Vill.	Leaves	ATCC 25175	2000	nt	+	[23]
*Baccharis dracunculifolia*	Leaves	UA 159	62.5–125	250–500	++++	[11]
*Bidens sulphurea*	Leaves	ATCC 25175	250	nt	+++	[23]
*Cinnamomum zeylanicum*	Leaves	UA 159	250–500	500–1000	+++	[11]
*Coriandrum sativum*	Leaves	UA 159	31.2–62.5	62.5–125	++++	[11]
*Croton cajucara* Benth	Leaves	ATCC 4646	40.1	13.8	++++	[21]
*Cryptomeria japonica*	aerial parts	ATCC 25175	100	200	++++	[20]
*Cuminum cyminum*	CS	PTCC 1601	4000	nt	−	[24]
*Cymbopogon citratus*	Leaves	UA 159	125–250	250–500	+++	[11]
*Cymbopogon martini*	leaves	UA 159	125–250	250–500	+++	[11]
*Cymbopogon winterianus*	Leaves	UA 159	125–250	250–500	+++	[11]
*Cyperus articulatus*	Bulbs	UA 159	125–250	250–500	+++	[11]
*Elyonurus muticus*	Leaves	UA 159	125–250	250–500	+++	[11]
*Eucalyptus radiate*	CS	JC-2	10,000	10,000	−	[25]
*Eugenia caryophyllata* L.	CS	ATCC 25175	200	800	+++	[26]
*Eugenia caryophyllata* L.	CS	ATCC 5175	600	nt	++	[27]
*Eugenia florida*	Leaves	UA 159	125–250	125–250	+++	[11]
*Eugenia uniflora*	Leaves	UA 159	125–250	250–500	+++	[11]
*Foeniculum vulgare* Mill.	Leaves	ATCC 25175	>4000	nt	−	[23]
*Lavandula officinalis*	CS	JC-2	>10,000	>10,000	−	[25]
*Leptosperfum scoparium*	CS	JC-2	2500	2500	−	[25]
*Lippia alba*	Leaves	ATCC 25175	500	nt	+++	[23]
*Lippia alba*	Leaves	UA 159	125–250	250–500	+++	[11]
*Lippia sidoides*	Leaves	UA 159	62.5–125	125–250	++++	[11]
*Melaleuca alternifólia*	CS	JC-2	10,000	10,000	−	[25]
*Melaleuca alternifólia*	Leaves	clinical isolates	0.25–2	0.25–2	*	[28]
*Mentha piperita*	Leaves	UA159	250–500	250–500	+++	[11]
*Mentha piperita*	CS	PTCC 1601	6000	nt	+	[24]
*Mikania glomerata*	Leaves	UA 159	62.5–125	125–250	++++	[11]
*Ocimum americanum* L.	Leaves	ATCC 6363	0.04	0.08	*	[29]
*Ocimum gratissimum* L*.*	Leaves	ATCC 25175	1000	nt	++	[23]
*Pelargonium graveolens*	Leaves	ATCC 25175	1000	nt	++	[23]
*Romarinus officinalis* L.	Leaves	JC-2	>10,000	>10,000	−	[25]
*Rosmarinus officinalis* L*.*	Leaves	ATCC 25275	>2000	nt	−	[15]
*Rosmarinus officinalis* L*.*	CS	PTCC 1601	2000	nt	−	[30]
*Satureja biflora*	flowering aerial parts	clinical isolates	640	nt	++	[31]
*Satureja masukensis*	flowering aerial parts	clinical isolates	570	nt	++	[31]
*Satureja pseudosimensis*	Leaves and flowering tops	clinical isolates	920	nt	++	[31]
*Siparuna guianenses*	Leaves	UA 159	62.5–125	125–250	++++	[11]
*Syzygium aromaticum*	Leaves	ATCC 25175	2000	nt	+	[23]
*Syzygium aromaticum*	Leaves	UA 159	250–500	250–500	+++	[11]
*Tagetes erecta* L.	Leaves	ATCC 25175	>4000	nt	−	[23]
*Thymus eriocalyx*	CS	PTCC 1601	2000	nt	+	[30]
*Zivuphus zoazeiro*	Leaves	UA 159	250–500	500–1000	+++	[11]

Note: * values are expressed as *v*/*v*; CS (commercial source); nt (not tested); Comparative MIC values: (++++) ≤100; (+++) 101 to 500; (++) 501 to 1000; (+) >1001 to 2000; (−) >2001.

##### Crude EOs and Planktonic *S. sobrinus*, *S. sanguinis* and *S. salivarius*

Four plant species were found to have very strong or strong antibacterial activity against *S. sobrinus*, as follows: *Croton cajucara* Benth (sacaca) [21]; *Rosmarinus officinalis* L. (rosemary) [15]; *Eugenia caryophyllata* L. (clove) [26] and *Cryptomeria japonica* (sugi) [20]. Of these, *C. japonica* also had very strong and strong activity against *S. sanguinis* and *S. salivarius*, respectively (Table 2).

##### Crude EOs and Planktonic Lactobacilli

*Achillea ligustica* (ligurian yarrow) [19] had strong activity against *L. acidophilus*. Another species of *Lactobacillus*, *L. casei*, was found to be strongly susceptible to *Croton cajucara* (sacaca) [21], *Artemisia camphorata* Vill. (camphor), *Bidens sulphurea* Sch. Bip. (yellow cosmos), *Lippia alba* Mill. (lemon balm) and *Ocimum gratissimum* L. (tree basil) [23] (Table 3).

##### EO-Isolated Compounds against Streptococci and Lactobacilli

Menthol, isolated from *Mentha longifolia* L., and eugenol, isolated from *Eugenia caryophyllata* L., were found to be the most promising compounds with strong activity against streptococci and lactobacilli (Table 4, Table 5 and Table 6).

**Table 2:** *In vitro* antibacterial activity of essential oils against *S. sobrinus*, *S. sanguinis* and *S. salivarius.*

Plant Species	Source	Microorg	*S. sobrinus* ^1^			*S. sanguinis* ^2^			*S. salivarius* ^3^			Ref.
			MIC (µg/mL)	MBC (µg/mL)	Score	MIC (µg/mL)	MBC (µg/mL)	Score	MIC (µg/mL)	MBC (µg/mL)	Score	
*Achillea ligustica* All	inflorescences	IMC104 ^3^	nt	nt		nt	nt		1250	nt	+	[19]
*Achillea ligustica* All	Leaves	IMC104 ^3^	nt	nt		nt	nt		1250	nt	+	[19]
*Achillea ligustica* All	flowering aerial parts	IMC104 ^3^	nt	nt		nt	nt		625	nt	++	[19]
*Ageratum conyzoides* L*.*	Leaves	ATCC 33478 ^1^	>4000	nt	−	>4000	nt	−	4000	nt	−	[23]
		ATCC 10556 ^2^										
		ATCC 25975 ^3^										
*Artemisia* *camphorata* Vill*.*	Leaves	ATCC 33478 ^1^	2000	nt	+	2000	nt	+	4000	nt	−	[23]
		ATCC 10556 ^2^										
		ATCC 25975 ^3^										
*Bidens sulphurea*	Leaves	ATCC 33478 ^1^	4000	nt	−	4000	nt	−	4000	nt	−	[23]
		ATCC 10556 ^2^										
		ATCC 25975 ^3^										
*Croton cajucara* Benth	Leaves	ATCC 27609 ^1^	13.8	nt	++++	nt	nt		nt	nt		[21]
*Cryptomeria japonica*	aerial parts	ATCC 27607 ^1^	100	100	++++	100	200	++++	nt	nt		[20]
		ATCC 10556 ^2^										
*Eucalyptus radiate*	CS	ATCC 6715 ^1^	10,000	10,000	−	nt	nt		nt	nt		[25]
		ATCC B13 ^1^										
*Eugenia caryophyllata* L.	Flowers	ATCC 27607 ^1^	200	800	+++	400	800	+++	nt	nt		[26]
		ATCC 10556 ^2^										
*Foeniculum vulgare* Mill.	Leaves	ATCC 33478 ^1^	>4000	nt	−	>4000	nt	−	>4000	nt	−	[23]
		ATCC 10556 ^2^										
		ATCC 25975 ^3^										
*Lavandula officinalis*	CS	6715 ^1^ B13 ^1^	10,000 10,000	10,000 10,000	− −	nt	nt		nt	nt		[25]
*Leptosperfum scoparium*	CS	6715 ^1^ B13 ^1^	1300 2500	2500 2500	+ −	nt	nt		nt	nt		[25]
*Lippia alba*	Leaves	ATCC 33478 ^1^	1000	nt	++	1000	nt	++	2000	nt	+	[23]
		ATCC 10556 ^2^										
		ATCC 25975 ^3^										
*Melaleuca alternifólia*	CS	6715 ^1^ B13 ^1^	10,000 2500	10,000 10,000	− −	nt	nt		nt	nt		[25]
*Mentha piperita*	CS	Ssb 176 ^1^	3000	nt	−	6000	nt	−	nt	nt		[32]
		Ssg 009 ^2^										
*Ocimum basilicum*	CS	Ssb 176 ^1^	6000	nt	−	6000	nt	−	nt	nt		[32]
		Ssg 009 ^2^										
*Ocimum gratissimum* L*.*	Leaves	ATCC 33478 ^1^	1000	nt	++	2000	nt	+	2000	nt	+	[23]
		ATCC 10556 ^2^										
		ATCC 25975 ^3^										
*Pelargonium graveolens*	Leaves	ATCC 33478 ^1^	1000	nt	++	2000	nt	+	2000	nt	+	[23]
		ATCC 10556 ^2^										
		ATCC 25975 ^3^										
*Rosmarinus officinalis* L*.*	Leaves	6715 ^1^	10,000 10,000	10,000 10,000	− −	nt	nt		nt	nt		[25]
		B13 ^1^										
*Rosmarinus officinalis* L*.*	Leaves	ATCC 33478 ^1^	500	nt	+++	>2000	nt	−	600	nt	++	[15]
		ATCC 10556 ^2^										
		ATCC 25975 ^3^										
*Salvia officinalis*	CS	Ssb 176 ^1^	3000	nt	−	6000	nt	−	nt	nt		[32]
		Ssg 009 ^2^										
*Syzygium aromaticum*	Leaves	ATCC 33478 ^1^	>4000	nt	−	>4000	nt	−	>4000	nt	−	[23]
		ATCC 10556 ^2^										
		ATCC 25975 ^3^										
*Tagetes erecta* L*.*	Leaves	Ssb 176 ^1^	6000	nt	−	nt	nt		nt	nt		[32]
		Ssg 009 ^2^										

Note: CS = commercial source; nt (not tested); Comparative MIC values: (++++) ≤100; (+++) 101 to 500; (++) 501 to 1000; (+) >1001 to 2000; (−) >2001; ^1^ *S. sobrinus*; ^2^ *S. sanguinis* and ^3^ *S. salivarius*.

**Table 3:** *In vitro* antibacterial activity of essential oils against lactobacilli.

Plant Species	Source	Microorg	*L. acidophilus* ^1^			*L. casei* ^2^			Ref.
			MIC (µg/mL)	MBC (µg/mL)	Score	MIC (µg/mL)	MBC (µg/mL)	Score	
*Achillea ligustica* All.	Inflorescences	IMC 101 ^1^	310	nt	+++	nt	nt		[19]
*Achillea ligustica* All.	Leaves	IMC 101 ^1^	2500	nt	−	nt	nt		[19]
*Achillea ligustica* All.	flowering aerial parts	IMC 101 ^1^	1250	nt	+	nt	nt		[19]
*Ageratum conyzoides* L.	Leaves	ATCC 11578 ^2^	nt	nt		4000	nt	-	[23]
*Artemisia camphorata* Vill.	Leaves	ATCC 11578 ^2^	nt	nt		500	nt	+++	[23]
*Bidens sulphurea*	Leaves	ATCC 11578 ^2^	nt	nt		500	nt	+++	[23]
*Croton cajucara* Benth	Leaves	ATCC 4646 ^2^	nt	nt		22.3	nt	++++	[21]
*Foeniculum vulgare* Mill.	Leaves	ATCC 11578 ^2^	nt	nt		4000	nt	−	[23]
*Lippia alba*	Leaves	ATCC 11578 ^2^	nt	nt		500	nt	+++	[23]
*Ocimum americanum* L.	Leaves	ATCC 6363 ^2^	nt	nt		0.04	0.3 *	*	[29]
*Ocimum basilicum*	aerial parts	ATCC 4356 ^1^	80,000	nt	−	nt	nt		[33]
*Ocimum gratissimum* L.	Leaves	ATCC 11578 ^2^	nt	nt		500	nt	+++	[23]
*Origanum vulgare*	aerial parts	ATCC 4356 ^1^	5000	nt	−	nt	nt		[33]
*Pelargonium graveolens*	Leaves	ATCC 11578 ^2^	nt	nt		1000	nt	++	[23]
*Rosmarinus officinalis*	aerial parts	ATCC 4356 ^1^	80,000	nt	−	nt	nt		[33]
*Salvia officinalis*	aerial parts	ATCC 4356 ^1^	80,000	nt	−	nt	nt		[33]
*Syzygium aromaticum*	Leaves	ATCC 11578 ^2^	nt	nt		1000	nt	++	[23]
*Tagetes erecta* L.	Leaves	ATCC 11578 ^2^	nt	nt		4000	nt	−	[23]
*Thymus vulgaris*	aerial parts	ATCC 4356 ^1^	5000	nt	−	nt	nt		[33]

Note: * values are expressed as % (*v*/*v*); nt (not tested); Comparative MIC values: (++++) <100; (+++) 100 to 500; (++) 501 to 1000; (+) >1001 to 2000; (−) >2001; ^1^ *L. acidophilus*; ^2^ *L. casei*.

**Table 4:** Essential oils isolated compounds against *Streptococcus mutans*.

Compound	Plant Species	Culture Collection	MIC (μg/mL)	MBC (μg/mL)	Score	Ref.
1,8, Cineole	*Achillea ligustica* All	DSM 20523	2500	nt	−	[19]
1,8, Cineole	*Achillea ligustica* All	DSM 20523	155	1250	+++	[22]
1,8, Cineole	*Rosmarinus officinalis*	ATCC 25275	1500	nt	+	[15]
Camphor	*Rosmarinus officinalis*	ATCC 25275	1500	nt	+	[15]
Caryophyllene oxide	*Satureja species*	clinical isolates	250	nt	+++	[31]
Eugenol	*Eugenia caryophyllata* L.	ATCC 25175	100	200	++++	[26]
Linalool	*Achillea ligustica* All	DSM 20523	625	nt	++	[19]
Linalool	*Achillea ligustica* All	DSM 20523	310	310	+++	[22]
Linalool	*Croton cajucara* Benth	ATCC 25175	no activity	nt	−	[21]
Linalool	*Satureja species*	clinical isolates	370	nt	+++	[31]
Menthol	*Mentha longifolia* L.	clinical isolates	15.6	nt	++++	[34]
Pulegone	*Satureja species*	clinical isolates	1750	nt	+	[31]
Sabinene	*Cryptomeria japonica*	ATCC 25175	800	1600	++	[20]
Terpinen-4-ol	*Achillea ligustica* All	DSM 20523	1250	nt	+	[19]
Terpinen-4-ol	*Achillea ligustica* All	DSM 20523	310	625	+++	[22]
Terpinen-4-ol	*Cryptomeria japonica*	ATCC 25175	1600	3200	+	[20]
Verbenone	*Rosmarinus officinalis*	ATCC 25275	1000	nt	++	[15]
Viridiflorol	*Achillea ligustica* All	DSM 20523	2500	nt	−	[19]
α-Pinene	*Cryptomeria japonica*	ATCC 25175	6400	28,000	−	[20]
α-Pinene	*Rosmarinus officinalis*	ATCC 25275	2000	nt	+	[15]
α-Terpineol	*Cryptomeria japonica*	ATCC 25175	1600	3200	+	[20]
β-Caryophyllene	*Eugenia caryophyllata* L.	ATCC 25175	1600	3200	+	[26]
β-Caryophyllene	*Rosmarinus officinalis*	ATCC 25275	300	nt	+++	[15]
β-Myrcene	*Rosmarinus officinalis*	ATCC 25275	400	nt	+++	[15]
β-Pinene	*Achillea ligustica* All	DSM 20523	1250	nt	+	[19]
β-Pinene	*Achillea ligustica* All	DSM 20523	625	1250	++	[22]
γ-Terpinene	*Achillea ligustica* All	DSM 20523	2500	nt	−	[19]

Note: CS (commercial source); nt (not tested); Comparative MIC values: (++++) < 100; (+++) 100 to 500; (++) 501 to 1000; (+) > 1001 to 2000; (−) > 2001.

**Table 5:** Essential oils isolated compounds against lactobacilli.

Compound	Source	Culture Collection	*L. acidophilus* ^1^			*L. casei* ^2^			Ref.
			MIC (µg/mL)	MBC (µg/mL)	Score	MIC (µg/mL)	MBC (µg/mL)	Score	
1,8, Cineole *	*Achillea ligustica* All	IMC101 ^1^	5000	nt	−	nt	nt		[19]
Linalool	*Croton cajucara* Benth	ATCC 4646 ^2^	nt	nt		no activity	nt	−	[21]
Linalool *	*Achillea ligustica* All	IMC101 ^1^	5000	nt	−	nt	nt		[19]
Menthol	*Mentha longifolia* L.	clinical isolates	31.2	nt	++++	nt	nt		[34]
Terpinen-4-ol *	*Achillea ligustica* All	IMC101 ^1^	5000	nt	−	nt	nt		[19]
β-Pinene *	*Achillea ligustica* All	IMC101 ^1^	2500	nt	−	nt	nt		[19]
γ-Terpinene *	*Achillea ligustica* All	IMC101 ^1^	5000	nt	−	nt	nt		[19]

Note: * standard from Sigma-Aldrich^®^ (St. Louis, MO, USA); nt (not tested); Comparative MIC values: (++++) <100; (−) >2001; ^1^ *L. acidophilus*; ^2^ *L. casei*.

**Table 6:** Essential oils isolated compounds against *S. sobrinus*, *S.* *sanguinis* and *S. salivarius.*

Compound	Plant Species	Culture Collection	*S. sobrinus* ^1^			*S. sanguinis* ^2^			*S. salivarius* ^3^			Ref.
			MIC (µg/mL)	MBC (µg/mL)	Score	MIC (µg/mL)	MBC (µg/mL)	Score	MIC (µg/mL)	MBC (µg/mL)	Score	
1,8-cineole	*Achillea ligustica* All	IMC104 ^3^	nt	nt		nt	nt		1250	nt	+	[19]
Camphor	*Rosmarinus officinalis*	ATCC 33478 ^1^	1500	nt	+	400	nt	+++	400	nt	+++	[15]
		ATCC 10556 ^2^										
		ATCC 25975 ^3^										
Eugenol	*Eugenia caryophyllata* L.	ATCC 27607 ^1^	200	400	+++	400	800	+++	nt	nt		[26]
		ATCC 10556 ^2^										
Linalool	*Achillea ligustica* All	IMC104 ^3^	nt	nt		nt	nt		625	nt	++	[19]
Linalool	*Croton cajucara* Benth	ATCC 27609 ^1^	no activity	nt	−	nt	nt		nt	nt		[21]
Sabinene	*Cryptomeria japonica*	ATCC 27607 ^1^ ATCC 10556 ^2^	200	200	+++	400	400	+++	nt	nt		[20]
Terpinen-4-ol	*Achillea ligustica* All	IMC104 ^3^	nt	nt		nt	nt		625	nt	++	[19]
Terpinen-4-ol	*Cryptomeria japônica*	ATCC 27607 ^1^	1600	3200	+	1600	3200	+	nt	nt		[20]
		ATCC 10556 ^2^										
Verbenone	*Rosmarinus officinalis*	ATCC 33478 ^1^	1000	nt	++	400	nt	+++	400	nt	+++	[15]
		ATCC 10556 ^2^										
		ATCC 25975 ^3^										
Viridiflorol	*Achillea ligustica* All	IMC104 ^3^	nt	nt		nt	nt		625	nt	++	[19]
α-Pinene	*Cryptomeria japonica*	ATCC 27607 ^1^	6400	12.800	−	6400	6400	-	nt	nt		[20]
		ATCC 10556 ^2^										
α-Pinene	*Rosmarinus officinalis*	ATCC 33478 ^1^	1000	nt	++	400	nt	+++	400	nt	+++	[15]
		ATCC 10556 ^2^										
		ATCC 25975 ^3^										
α-Terpineol	*Cryptomeria japônica*	ATCC 27607 ^1^	1600	1600	+	1600	3200	+	nt	nt		[20]
		ATCC 10556 ^2^										
β-Caryophyllene	*Eugenia caryophyllata* L.	ATCC 27607 ^1^	12,800	12,800	−	1600	3200	+	nt	nt		[26]
		ATCC 10556 ^2^										
β-Caryophyllene	*Rosmarinus officinalis*	ATCC 33478 ^1^	400	nt	+++	400	nt	+++	400	nt	+++	[15]
		ATCC 10556 ^2^										
		ATCC 25975 ^3^										
β-Myrcene	*Rosmarinus officinalis*	ATCC 33478 ^1^	1500	nt	+	1500	nt	+	400	nt	+++	[15]
		ATCC 10556 ^2^										
		ATCC 25975 ^3^										
β-Pinene	*Achillea ligustica* All	IMC104 ^3^	nt	nt		nt	nt		625	nt	++	[19]
γ-Terpinene	*Achillea ligustica* All	IMC104 ^3^	nt	nt		nt	nt		625	nt	++	[19]

Note: nt (not tested); Comparative MIC values: (+++) 100 to 500; (++) 501 to 1000; (+) >1001 to 2000; (−) >2001; ^1^ *S. sobrinus*; ^2^ *S. sanguinis* and ^3^ *S. salivarius*.

#### 2.1.2. Biofilm Studies

##### Crude EOs and Biofilms of Streptococci and Lactobacilli

A total of eight species were tested against biofilm cultures of *S. mutans*, *S. sobrinus* and/or *L. casei* using different assays (Table 7). Interestingly, bioactive fractions of *C. sativum* and *B. dracunculifolia* inhibited 90% of *S. mutans* biofilm formation at concentrations as low as 31.2 μg/mL. Moreover, *C. cajucara* EO (100 μg/mL) and *O. americanum* EO (3%) inhibited *S. mutans* and *L. lactis* biofilms as effectively as chlorhexidine, used as positive control.

Overall, the majority of studies in this review tested the effectiveness of EO against *S. mutans* (35 out of 40 studies), followed in lower proportions by *S. sobrinus*, *S. salivarius*, *S. sanguinis* and *Lactobacillus* spp. As seen in Table 8, just a few studies carried out a comprehensive analysis of the effect of EO against a broad panel of caries-related species.

### 2.2. In Vivo Studies

#### Randomized Clinical Trials

**Figure 2:**
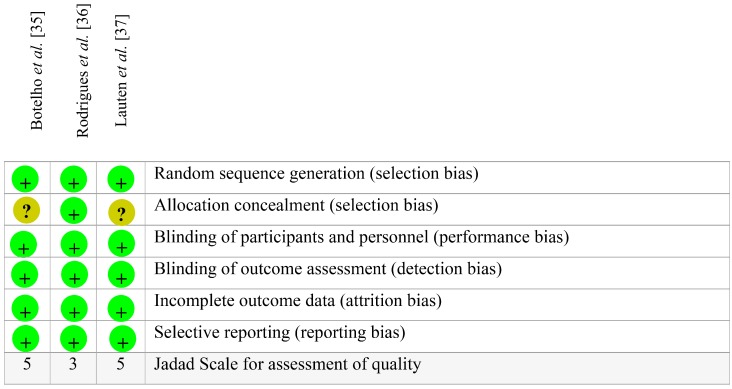
Risk-of-bias summary of the clinical trials included in this systematic review. Red (−) stands for high risk of bias, green (+) stands for low risk of bias and yellow (?) stands for unclear risk of bias. Overall, the studies are compliant with the CONSORT guidelines for clinical trials of herbal interventions, showing low risk of bias.

Three high quality randomized, double-blind clinical trials of herbal interventions with low risk of bias were included in this review (Figure 2). The EOs from *L. sidoides* [35,36] and a multi-herbal formulation including *Melaleuca alternifolia* and *Leptospermum scoparium* oils (combined with *Calendula* *officinalis* and *Camellia sinensis* extracts) [37], were tested in humans for their effectiveness in reducing the amount of cariogenic biofilm, measured by means of plaque indexes. The experimental period of studies ranged from 1 week to 12 weeks, with different assessment checkpoints and dosing protocols. As seen in Table 9, only individuals treated with 1% *L. sidoides* EO mouthwash had a statistically significant reduction in their supragingival biofilm levels compared to chlorhexidine group (positive control) and to their baseline condition.

### 2.3. Chemical and Botanical Characterization and Georeferencing of the most Promising Bioactive EOs

Viridiflorol, terpinen-4-ol and β-pinene are found in the EO from all parts [19,22] of *A. lingustica*; however, important terpenes such as linalool, 1,8-cineole and germacrene D have also been identified in specific parts of the plant. Elemol, terpinen-4-ol, sabinene, 10(15)-cadinen-4-ol, α-terpineol and α-pinene are the major compounds identified in *C. japonica* [20]. Linalool is the most abundant compound of *C.* *cajucara* Benth [21]. Trans-nerolidol, spathulenol and trans-caryophyllene are found in *B. dracunculifolia* [11]. 1-decanol, trans-2-decen-1-ol and 2-dodecen-1-ol are the most abundant compounds of *C. sativum* [11]. Thymol is the major compound of *L. sidoides* [11]. Camphor, verbenone, α-pinene, β-myrcene, 1,8-cineole and β-caryophyllene are found in *R. officinalis* [15]. Eugenol and β-caryophyllene are the major compounds of *E. caryophyllata* [26]. 

**Table 7:** Essential oils, fractions or isolated compounds against *in vitro* oral biofilm formation.

Ref.	Essential Oil/Fraction or Isolated Compound	Biofilm Formation			
		Strain	Test(s) Performed	Biofilm Age Conditions	Outcomes
[11]	*Aloysia gratíssima* (Ag), *Coriandrum sativum* (Cs) and *Baccharis dracunculifolia* (Bd) fraction	*S. mutans UA159*	Formation of S. mutans biofilm, the samples were placed in the wells of sterile polystyrene U-bottom microtiter plates, previously treated with saliva	*S. mutans* cells (1.0 × 10^7^ cells/mL in BHI medium) were added to wells containing BHI medium with 2% sucrose and the samples were incubated at 37 °C for 18 h	Biofilm of Cs4 and Bd2 fractions presented a better performance since they inhibited more than 90% of biofilm formation at lower concentrations (31.2 μg/mL).
[21]	*Croton cajucara* Benth leaves	*S. mutans* ATCC 25175	Macro technique using microbial disks subjected to the action of the essential oil and controls	The biofilms were exposed to controls and essential oil for 3 min and incubated for 72 h at 37 °C	Growth inhibition: EO 70%–75% Chlorhexidine 65%–70%
[21]	*Croton cajucara* Benth leaves	*S. sobrinus* ATCC 27609	Macro technique using microbial disks subjected to the action of the essential oil and controls	The biofilms were exposed to controls and essential oil for 3 min and incubated for 72 h at 37 °C	Growth inhibition: EO 75%–80% Chlorhexidine 50%–55%
[21]	*Croton cajucara* Benth leaves	*L. casei* ATCC 4646	Macro technique using microbial disks subjected to the action of the essential oil and controls	The biofilms were exposed to controls and essential oil for 3 min and incubated for 72 h at 37 °C	Growth inhibition: EO 80%–85% Chlorhexidine 65%–70%
[38]	*Curcuma longa* root	*S. mutans* ATCC 25175	Technique using 24-well plates containing resin teeth.	After cultivating *S. mutans* for 24 h at 37 °C, the supernatant was removed, and the wells were rinsed with distilled H_2_O. Biofilm formation in the wells was measured by staining with 0.1% safranin	Biofilm formation was decreased in the presence of *C. longa* essential oil at concentrations higher than 500 µg/mL
[39]	*Mentha piperita and Rosmarinus officinalis*	*S. mutans* PTCC 1601	Biofilm formation (SBF) assay	The biofilms were exposed to controls and essential oil and incubated for 17 ± 1 h at 37 °C	*M. piperita* and *R. officinalis* oils effectively inhibited *S. mutans* biofilm at 6000 and 2000 ppm, respectively.
[29]	*Ocimum americanum* L. leaves	*S. mutans* KPSK2	Microtiter technique Protocol using saliva.	The biofilms were exposed to controls and essential oil (0.3% and 3% *v*/*v*) for 5 min and incubated for 24 h	EO 0.3% (*v*/*v*) 7.2 × 10^4^ CFU/mL; EO 3% (*v*/*v*) 2.9 × 10^3^ CFU/mL; 0.2% Chlorhexidine: 1.7 × 10^3^ CFU/mL; Saline solution 8.5.10^6^ CFU/mL
[29]	*Ocimum americanum* L. leaves	*L. casei* ATCC 6363	Microtiter technique Protocol using saliva.	The biofilms were exposed to controls and essential oil (0.3% and 3% *v*/*v*) for 5 min and incubated for 24 h	EO 0.3% (*v*/*v*) 5.1 × 10^5^ CFU/mL; EO 3% (*v*/*v*) 6.3 × 10^3^ CFU/mL; 0.2% Chlorhexidine: 2.5 × 10^3^ CFU/mL; Saline solution 6.0 × 10^6^ CFU/mL

**Table 8:** Framework of studies. Distribution of promising EOs and their isolated constituents tested against caries-related bacteria.

Plant Species or Chemical Constituent	Antibacterial Efficacy										
	Planktonic Cells						Biofilms				Clinical Trial
	Smu	Ssob	Ssan	Ssal	Lc	La	Smu	Ssob	Ssal	Lc	
*A. ligustica*	+										
*B. dracunculifolia*	+										
*C. cajucara*	+	+			+		+	+		+	
*C. japonica*	+	+	+								
*C. sativum*	+										
*E. caryophyllata*	+	+	+								
*L. sidoides*	+										Plaque reduction
*O. americanum*	+				+		+			+	
Menthol	+					+					
Eugenol	+	+	+								

Note: (+): MIC <100 µg/mL or correspondent; Smu: *S. mutans*; Ssob: *S. sobrinus*; Ssan: *S. sanguinis*; Ssal: *S. salivarius*; Lc: *L. casei*; La: *L. acidophilus*.

**Table 9:** Characteristics of the Randomized Clinical Trials included in this systematic review.

Plant Species	Essential Oil Formulation	Study Design	Sample Size	Country	Age (Mean ± SD)/Gender (Fem) *	Sample Loss/Reasons	Control Group	Dosing Protocol	Assessment Checkpoints	Assessment Instruments of Interest	Outcome **	Ref.
*Lipia sidoides*	1% *L. sidoides* mouthrinse	Phase II, randomized, double-blind, crossover	*n* = 55 (*n* = 27 *L. sidoides* group; *n* = 28 control group)	Brazil	31 ± 10.90 55.6% F	16 individuals (no gender distinction)/lack of compliance or could not be reached for follow-up visits.	0.12% CHX	Rinsing approx. 15 mL for 30 s, twice a day (once after breakfast and once in the late afternoon) during a 7-day period.	Baseline, 1 week	Plaque index (PI) measured at four sites *per* tooth (Ainamo & Bay, 1975)	+/+	[35]
*Lipia sidoides*	10% *L. sidoides* gel	Phase II, randomized, double-blind, crossover. Partial mouth experimental model	*n* = 26 (*n* = 13 *L. sidoides* group; *n* = 13 control group)	Brazil	22 ± 4.24 50.0% F	4 individuals (no gender distinction)/third molar extraction	Placebo gel	Filling a toothshield with the gel prior to insertion in the mouth and seating it over the experimental teeth 3 times a day for at least 1 min.	Baseline, 3 weeks	Plaque index (PI) measured at six sites *per* tooth (Turesky *et al.*, 1970)	−/+	[36]
*Melaleuca alternifolia*, *Leptospermum scoparium*, *Calendula officinalis* and *Camellia sinensis*	Multi-herbal mouthrinse containing 0.67% (*v*/*v*) *M. alternifolia* oil, 0.33% (*v*/*v*) *M. scoparium* oil, 1% (*v*/*v*) *C. officinalis* flower extract (1:2) liquid extract [90% E/W]), 0.5% (*w*/*v*) *C. sinensis* extract (dry extract, 80% polyphenols) and 12.8% ethanol in water.	Phase I and II, randomized, double-blind	Phase I *n* = 8 (experimental group) Phase II *n* = 20 (*n* = 10 experim. group; *n* = 10 control group)	USA	31.88 ± 7.51 Phase I: 62.5% F Phase II: 82.3% F	Phase I: 1 female/reported mild ‘hay fever’-like symptoms. Her symptoms were judged to be unrelated to the mouthrinse. Phase II: 3 female/One reported lightheadedness (possibly related to the test rinse); One dropped out to participate in another study; and one was excluded because she required treatment with antibiotics for an unrelated condition.	Placebo mouth rinse	Rinsing approx. 15 mL for 30 s, twice a day during a 6-week period.	Baseline, 6 weeks and 12 weeks	Plaque index (PI) measured at six sites *per* tooth (Quigley & Hein, 1962)	−/−	[37]

Note: CHX (chlorhexidine) mouthrinse; * Age and gender of individuals assigned to the experimental group; ** Statistically significant reduction (+) or not (−) in the amount of cariogenic biofilm compared to CHX or placebo (fist sign) and to the baseline condition (second sign, after slash) (*p* < 0.05).

The EO from *A. camphorata*, *B. sulphurea*, *L. alba*, *M. glomerata*, *O. gratissimum* and *S. guianenses* were not chemically characterized by the studies included in this review. Therefore, 21.7% of the selected studies had no chemical control regarding the EO under test. Furthermore, only 60.8% of the studies proceeded with a botanical identification of the aromatic plants that served as source for the EO. Finally, only 56.52% of the studies showed any piece of information about georeferencing of the plant species and 47.82% reported the period of plant collection.

## 3. Discussion

Essential oils have stood out as a promising source of bioactive molecules with potential application in the management of dental caries [40,41]. The data presented in this review suggest potential EO and constituents to be further tested as bioactive ingredients of anti-caries formulations. Moreover, the results of the reported chemical assessments of EO-isolated compounds could lead them to be used as chemical markers in future screening. Surprisingly, 20% and 60% of the studies do not provide any chemical or botanical information, respectively, which inevitably results in a biased and inconclusive analysis with reproducibility and traceability issues. Also, despite an understanding of the biological and physicochemical processes associated with the aetiopathogenesis of dental caries [8], great part (88%) of the current evidence on the anti-caries potential of EO is based on *in vitro* studies rather than clinical trials (see Section 3.3 in this Discussion). Altogether, the benefits and issues related to EO research suggest wide avenues for scientists to work on more comprehensive and trustworthy bioprospection studies.

According to our searches, the majority of *in vitro* studies have evaluated the effect of EO or isolated compounds against *S. mutans*, as expected. Considered the most cariogenic of the oral streptococci, *S. mutans* colonizes the tooth surfaces and produces significant amounts of extra- and intra-cellular polysaccharides [42], being responsible for the initial stage of oral biofilm formation and carious lesions [43]. Nevertheless, other streptococci and lactobacilli species are also implicated on the onset [44] and progression [45] of caries, respectively, thus playing a role in the aetiopathogenesis of this biofilm-dependent disease. An EO of interest to be included in a formulation should be that able to affect bacterial virulence without suppressing the resident oral species, as a more specific therapeutic approach [8]. However, most studies provide just preliminary evidence of anti-caries activity without further assessing the effects of EO on putative virulence factors in cariogenic bacteria (e.g., glycosyltransferase and F-ATPase activity). In addition, the cariogenic biofilm is composed of a multi-species microbial community, in which the predominance of different microorganisms is changed as a function of host, diet and microorganism factors [46]. These aspects are not considered in most studies evaluating only planktonic cultures and, at most, monospecies biofilm cultures.

Next, we provide a brief summary of the plant species whose EO and their isolated compounds were found to have significant *in vitro* anti-caries potential. Attention is given to the ethnopharmacological knowledge, biological properties and chemical composition. Despite our attempts to make inter-study comparisons, there are underlying distinctions related to extraction methods, georeferencing, seasonality, which should be taken into account.

### 3.1. Promising Essential Oils against Cariogenic Bacteria

*Achillea ligustica* (Asteraceae) is a small herbaceous plant rich in terpenes that grows in the Mediterranean region and has been used in folk medicine mainly for the treatment of gastrointestinal disorders [47]. The EO from different parts of this plant (inflorescences, leaves and flowers) is also found to have antimicrobial activity, particularly against *S. mutans* [19,22]. However, as it can be seen in this review, when the major compounds of *A. ligustica* EO are tested alone (e.g., γ-terpinene, β-pinene, 1,8-cineole, terpinen-4-ol), there is a decrease in their antimicrobial activity, which suggests a synergistic effect of the compounds present in the whole EO. Different EOs from the genus *Achillea* have been used in the cosmetic and liqueur industry as fragrances and flavoring agents, demonstrating commercial and economic relevance [22].

*Baccharis dracunculifolia* (Asteraceae) a native plant from Brazil, is widespread in the tropical areas of South America and is the botanical source of Southeastern (or green) propolis [48]. It has been widely used in folk medicine as febrifuge, anti-inflammatory, antiseptic and in the treatment of skin sores and gastrointestinal disorders [49]. The *trans*-nerolidol- and spathulenol-rich EO from *B.* *dracunculifolia* and its active fractions are bacteriostatic and have an *in vitro* anti-cariogenic activity by disrupting *S. mutans* biofilm at concentrations as low as 31.25 µg/mL [11].

*Croton cajucara* (Euphorbiaceae) is a common shrub growing in the Amazonian region commonly used in folk medicine as a tea for ailments such as diarrhea, diabetes and gastrointestinal disorders [50]. Alviano *et al.* [21] found that the EO of *C. cajucara* has significant antibacterial activity against *S. mutans*, *S. sobrinus* and *L. casei* in planktonic and monospecies biofilm cultures, unlike its isolated major compound linalool. This result disagrees with others reported in this review showing that linalool is considerably active against *S. mutans* [19,22,31]; however, it remains controversial.

*Cryptomeria japonica* (Cupressaceae) is an endemic and widely distributed coniferous plant in Japan, normally used for forestry, whose EO has been reported to have several pharmacological properties including larvicidal [51], antiulcer [52], antifungal [53] and antibacterial [20]. *C. japonica* EO is another example of how the complex mixture of chemical molecules plays a synergistic role in the antibacterial power of the EO over its isolated major compounds (sabinene, terpinen-4-ol, α-pinene and α-terpineol) [20]. In this review, we found significant inhibitory effects of the leaf EO against caries-related streptococci, warranting further investigation.

*Coriandrum sativum* (Apiaceae) popularly known as coriander, is an annual small plant whose leaves and seeds are widely used in folk medicine as anti-hypertensive, cholesterol-lowering and digestive stimulant [54], and also as food condiment. Moreover, other biological properties of *C. sativum* EO have also been reported: antifungal [55,56] antibacterial [11,56], antioxidant [57] and hepatoprotective [58]. The EO from *C. sativum* leaves contains mostly decanal, *trans*-2-decenal and 2-decen-1-ol [55], and has been shown to have *in vitro* anti-cariogenic potential against *S. mutans* biofilms and to be more active than its chemical fractions [11].

*Eugenia caryophyllata* (Myrtaceae) is widely cultivated in Indonesia, Sri Lanka, Madagascar, Tanzania and Brazil. *E*. *caryophyllata* EO (clove) has been described as having useful antiseptic, analgesic and anaesthetic effects. In community medicine, it serves as a topical pain-relieving and healing agent and in the industry as a fragrance and flavoring substance [59]. The main compounds of clove oil are phenylpropanoids such as eugenol and β-caryophyllene. According to our findings, eugenol was proven to be more active than the EO against *S. mutans*, *i.e.*, showed lower MIC values. Nevertheless, the crude EO of E. *caryophyllata,* in general, showed strong antimicrobial activity against streptococci.

*Lippia sidoides* (Verbenaceae) is a typical shrub commonly found in the Northeastern Brazil, popularly used as topic skin and mucosal antiseptic [60]. *L. sidoides* EO also has anti-inflammatory, antioxidant and gastroprotective properties [61]. Its antimicrobial activity against cariogenic bacteria has been correlated with the presence of the phenolic monoterpenes thymol and carvacrol [62], and it may be considered of the most scientifically explored medicinal plants in Brazil, whose studies have reached the clinical phase. According to this review, *L. sidoides* EO showed both strong *in vitro* antibacterial activity and clinical efficacy as a mouthwash (see Section 3.3 in this Discussion), thus being considered a promising anti-plaque and anti-gingivitis phase II agent [37].

*Ocimum americanum* (Lamiaceae) popularly known as hoary basil, is an annual herbaceous plant native to Asia and Africa. *O. americanum* EO is reported to have anti-inflammatory, antinociceptive [63], antibacterial and insecticidal properties [64], and it is considered valuable for the cosmetic industry of soups and perfumes. The findings of this review showed that the leaf EO has strong antimicrobial activity against *S. mutans* and *L. casei*, either planktonic or biofilm cultures. The study by Thaweboon and Thaweboon [29] indicated that the 3% leaf EO is as effective as 0.2% chlorhexidine in reducing the bacterial counting of cariogenic biofilm cultures of *S. mutans* and *L. lactis*, thus highlighting its potential as an antiseptic agent for oral care. Other studies *in vitro* and *in vivo* are now encouraged to elucidate its effects on other aspects related to the aetiopathogenesis of tooth decay (e.g., glucosyltransferase activity, acid production, enamel demineralization, among others).

*Rosmarinus officinalis* (Lamiaceae) is a culinary evergreen shrub native to the Mediterranean region that has also been used for medicinal purposes to treat bacterial and fungal infections [65]. Unlike the other cases presented thus far, the major compounds of *R. officinalis* EO (camphor, verbenone, α-pinene, β-myrcene, 1,8-cineole and β-caryophyllene) showed better activity (lower MIC value) against cariogenic bacteria—particularly *S. sobrinus* and *S. salivarius*—than the crude EO.

### 3.2. Promising Compounds Isolated from Essential Oils against Cariogenic Bacteria

Generally, the major phytochemical compounds determine the biological properties of EOs [66]. In these cases, the study of isolated compounds is meaningful to concentrate the active principle, enable industrial scale production and allow improvements in the chemical structure using molecular engineering approaches. Here, we provide a summary on menthol and eugenol as the most outstanding compounds isolated from EOs that possess an anti-caries potential. 

**Figure 3:**
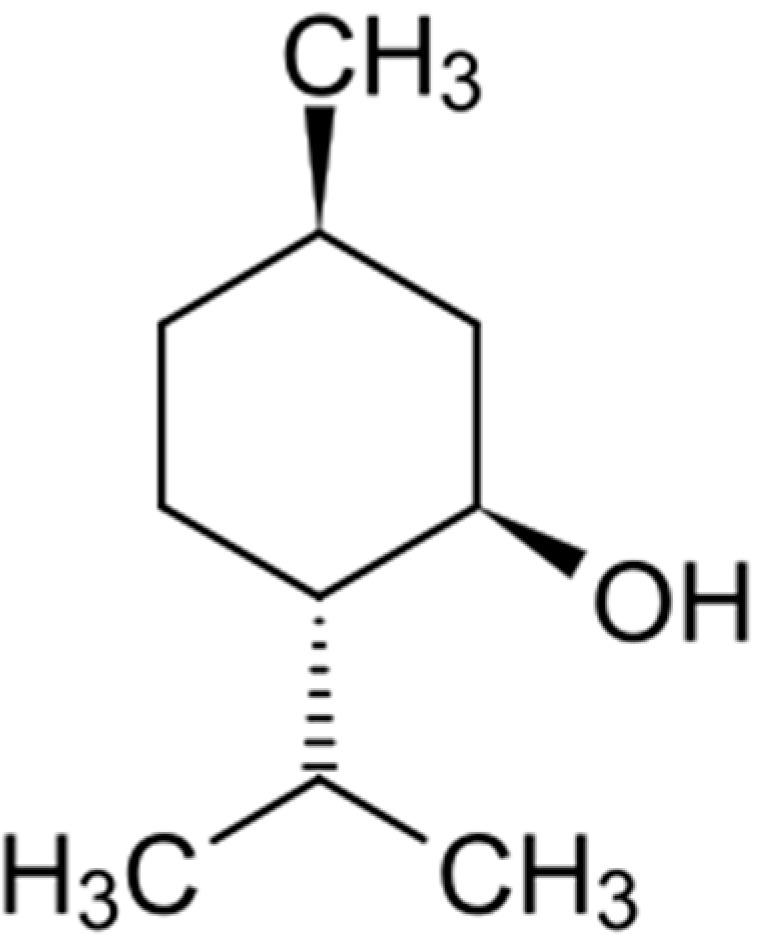
The chemical structure of (‒)-menthol [(1*R*,2*S*,5*R*)-2-isopropyl-5-methylcyclohexanol].

Menthol is a compound that has raised interest of the pharmaceutical and food industry in the last decades. It is a terpenoid that can be found in the EO of the *Mentha* spp. genus, such as peppermint, with a crystalline, clear or white-colored aspect (Figure 3). Although there are several isomers of menthol available, only (‒)-menthol occurs in nature [34]. 

*In vitro* [34,67] and *in situ* [68] studies have demonstrated that menthol inhibits the growth of both Gram-positive and -negative bacteria and yeasts, and that its mechanism of action may be related to membrane disruption leading to cell leakage. A number of clinical trials [18] have also supported the use of this compound as an ingredient of mouthwash formulations; some of which are already commercially available worldwide. Although menthol has been used more as a flavoring agent than an active principle, it has been proven to have a considerable antimicrobial activity and is considered as GRAS (*Generally Regarded as Safe*) by the FDA (US Food and Drug Administration).

**Figure 4:**
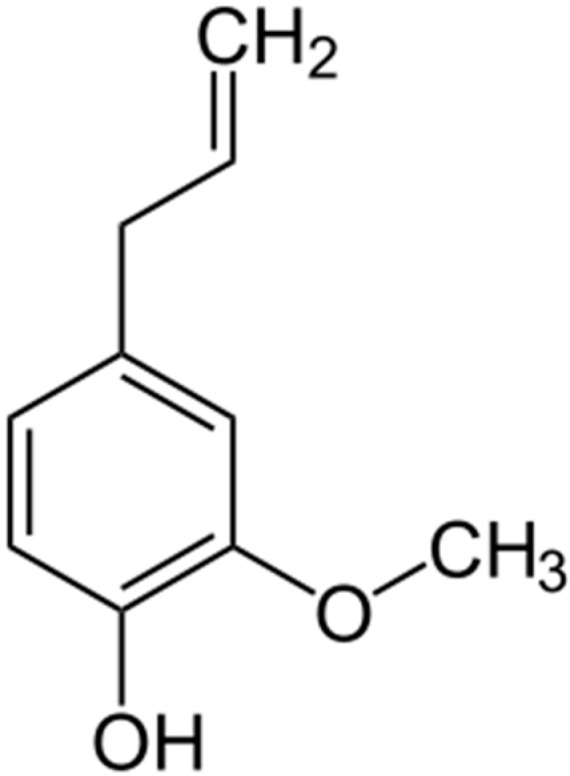
The chemical structure of eugenol [4-Allyl-2-methoxyphenol].

Eugenol is an amphipathic phenolic compound (Figure 4) representing the major constituent of EO from clove (*Eugenia caryophillis*) and cinnamon (*Cinnamomum zeylanicum*) leaves [12]. Eugenol has been reported to have antiseptic, antimicrobial, anesthetic, analgesic, antioxidant, anti-inflammatory, and cardiovascular activities [69]. In dentistry, it is used as component of a cement containing zinc oxide for provisional sealing of cavities or as base for definitive fillings [70]. According to our review, eugenol has a promising antimicrobial activity against streptococci, particularly *S. mutans*, and should be considered as an anti-cariogenic agent to further clinical testing. It is an interesting source of new drugs as it is classified as GRAS by the FDA. This compound has been commercially marketed.

In addition to these three compounds, some others indicated in this review arouse attention for their antibacterial power with MIC values lower than 500 µg/mL, as follows: 1,8, cineole, terpinen-4-ol, linalool, β-myrcene, β-caryophyllene and caryophyllene oxide. As such, the presence of these compounds in the EO of a plant could predict its antibacterial properties.

### 3.3. Rational Clinical Use of Essential Oils and Isolated Compounds

Despite the large number of *in vitro* studies on the antimicrobial activity of EOs, just a few reach the clinical phase and even fewer lead to a commercial product. Indeed, there is a small number of clinical trials reported in the literature aiming at the development of an EO-containing dental formulation.

The most effective way to use the majority of EOs is by external application, such as mouthwashes for dental care. Topical application is generally safe [66] because most compounds are considered as GRAS by the FDA and have been long used in food preparation in several cultures. In case of eventual oral administration of a mouthwash, for instance, most EO compounds (such as (−)-menthol, thymol, carvacrol and eugenol) would be excreted renally or exhaled via the lungs [71,72], and their fast metabolism and short half-life highlight a minimal risk of accumulation in the organism [73]. However, although EOs have the advantage of being usually devoid of long-term cytotoxicity and genotoxic risks [12], the high volatility and chemical instability of some of their compounds in the presence of heat, humidity, light, or oxygen, may negatively impact their clinical use [74].

At the present time, the most popular EO-based formulation used in dental care in Western society is composed of a fixed combination of four EO-derived active ingredients: thymol (0.064%), eucalyptol (0.092%), methyl salicylate (0.060%) and menthol (0.042%). It is considered effective against cariogenic bacteria and relatively safe, although its 21%–27% alcoholic formula used to keep the constituents in solution is still controversial [75]. In some cases, such as with *A. ligustica* [19,22], *C. japonica* [20] and *C. sativum* [56], the synergism of compounds in the EO is critical for its biological properties as opposite to its isolated constituents. Such chemical complexity may favor solubility in vehicles other than ethanol (e.g., propylene glycol), with less likelihood of adverse effects.

According to our analysis, the mouthwash of thymol- and carvacrol-rich *L. sidoides* EO (ethanol-free) rinsed twice a day is an effective agent to prevent/disrupt the accumulation of cariogenic biofilm [36]. Furthermore, in a previous systematic review [76] we also found that such experimental mouthwash was effective against biofilm-induced gingivitis in adults. Altogether, these findings highlight the therapeutic potential of *L.* *sidoides* EO for dental care, but it is important to note that further studies are needed to investigate its effects on other aspects related to tooth decay, such as bacterial acid production, biofilm formation, enamel de- and remineralization, inhibition of glycosyltransferase production/activity, among others. Furthermore, the 10% gel of thymol- and carvacrol-rich *L. sidoides* EO was not effective to reduce the amount of biofilm in adults compared to a placebo [37], suggesting that the pharmaceutical preparation plays a crucial role in this clinical outcome.

The synergistic association of EOs with other topical agents, e.g., fluoride, should also be considered for the management of dental caries, combining both antimicrobial and remineralization properties. A study by Zero *et al.* [77] showed that an EO mouthrinse with 100 parts per million fluoride should be effective in promoting enamel remineralization and fluoride uptake, thus providing anti-caries efficacy.

In dentistry, EOs could be useful as preoperative rinses, in periodontal procedures (e.g., sub-gingival irrigation), post-treatment applications, as a conventional mouthwash *etc.* Nevertheless, the majority of studies in the literature up to date fail to indicate robust and translational data to support the clinical use of novel EOs as ingredients of dental formulations, particularly against dental caries. With that said, this review suggests further research on the EOs and their constituents described earlier due to their favorable potential against streptococci and lactobacilli. In addition, it is important to determine the effects of EO on bacterial virulence factors related to dental caries, such as synthesis of extracellular polysaccharides and ability to survive in and produce acidic environments [8]. The scientific validation of the anti-caries activity of EOs and isolated compounds could provide not only patentable preparations and advances in preventive dentistry, but also commercial value.

## 4. Methods

### 4.1. Focused Question

The aim of the present review was to answer the specific question: “Based on the current literature, which essential oils and/or isolated compounds are promising anti-caries agents warranting further investigation for clinical use?”

### 4.2. Search Strategy and Selection of the Studies 

This systematic review of scientific studies followed the guidelines of the *Transparent Reporting of Systematic Reviews and Meta-Analyses (PRISMA statement)* [78]. Seven databases were systematically searched for clinical trials and *in situ*, *in vivo* and *vitro* studies (Table 10).

**Table 10:** Search strategy and bibliographic databases used to retrieve the articles falling into the scope of this systematic review.

Bibliographic Databases (Primary Sources)	Search Strategy (Descriptors and Boolean Operators)
SciVerse Scopus (Since 1995 until December 2014)	(oils, volatile OR essential oil) AND (anti caries OR anti caries agents) (oils, volatile OR essential oil) AND (mouthwashes OR dentifrice OR gel) AND anti plaque (oils, volatile OR essential oil) AND (oral pathogens OR cariogenic bacteria) (oils, volatile OR essential oil) AND antimicrobial AND oral cavity essential oils AND oral
Web of Science *(Refine: article or review)* (Since 1990 until December 2014)	(oils, volatile OR essential oil) AND (anti caries OR anti caries agents) (oils, volatile OR essential oil) AND (mouthwashes OR dentifrice OR gel) AND anti plaque (oils, volatile OR essential oil) AND (oral pathogens OR cariogenic bacteria)
Medline via Pubmed (Since 1966 until December 2014)	(oils, volatile OR essential oil) AND (anti caries OR anti caries agents) (oils, volatile OR essential oil) AND (mouthwashes OR dentifrice OR gel) AND anti plaque (oils, volatile OR essential oil) AND (oral pathogens OR cariogenic bacteria) (oils, volatile OR essential oil) AND antimicrobial AND oral cavity essential oil AND oral cavity AND antibacterial essential oil AND MIC AND oral
SciELO (Scientific Electronic Library Online) (Since 1998 until December 2014) and LILACS (Latin American and Caribbean Health Sciences Literature) (Since 1982 until December 2014)	aceites esenciales aceite volatile essential oil AND caries óleo essencial AND *Streptococcus mutans* óleo essencial AND *Lactobacillus* óleo essencial AND oral óleo essencial AND antibacteriano
Cochrane Library	essential oil AND caries óleo essencial AND *Streptococcus mutans* óleo essencial AND *Lactobacillus* óleo essencial AND oral óleo essencial AND antibacteriano
Google Scholar	Manual searches according to the reference lists of the articles

### 4.3. Eligibility Criteria

A systematic selection of the articles was carried out by three independent examiners based on the following inclusion criteria: (1) Biological activity: anti-caries activity against oral microorganisms involved in the etiology and progression of dental caries; (2) Plant material and chemical assessment: essential oils and/or isolated compounds from aromatic plants (their chemical assessment was not a restricted inclusion criteria, instead, it served as a point for discussion); (3) Study design: *In vitro*, *in situ* and/or *in vivo* laboratorial studies (planktonic and biofilm assays); randomized controlled clinical trials (outcome of interest: reduction in the amount of cariogenic biofilm); (4) Methodological quality: For clinical trials, *Jadad* scale [79] equal to or greater than 3, meeting high quality standards (see Section 4.4 for details); accuracy of outcomes; internal and external validity; (5) Language: Articles written in English, Spanish or Portuguese; (6) Novelty: Novel essential oils-containing dental formulations were included, if not currently marketed. Examiners agreed that in cases of inconsistence the final verdict on which articles should be included in this review would be reached by consensus.

### 4.4. Data Pooling and Analysis

The data were allocated into worksheets to proceed with exploratory analysis according to the study design. For *in vitro* studies, in order to standardize the susceptibility patterns of microorganisms to essential oils or isolated compounds, we used their minimum inhibitory concentration (MIC) range as a parameter to determine the intensity of antibacterial activity, based on the literature [80] and on our research experience (Table 11). The retrieved data were expressed according to the bacterial species related to different types of tooth decay, in terms of selectivity to specific surfaces: *Streptococcus mutans* (sulcus and fissure, smooth surface caries—main etiological agent of dental caries) [81]; *S. sanguinis*, *S. sobrinus*, *S. salivarius* play a secondary role and may be recovered from sulcus, fissure and smooth surface caries [82]; *Lactobacillus* spp. (dentin and root surface caries) [45], either in planktonic or biofilm assays.

**Table 11:** Established parameters based on Minimum Inhibitory Concentrations of essential oils or related chemical constituents.

MIC Range	Intensity of Antibacterial Activity	Score
≤100 µg/mL	very strong activity	(++++)
101–500 µg/mL	strong activity	(+++)
501–1000 µg/mL	moderate activity	(++)
1001–2000 µg/mL	weak activity	(+)
>2001 µg/mL	no activity	(−)

For clinical trials, the data were analyzed based on the CONSORT guidelines for reporting randomized, controlled trials of herbal interventions [83]. Jadad Scale [79] has also been adopted in this review as it checks the validity of evidence on interventions and evaluates methodological quality (randomization, blinding and loss of follow-up). Based on these criteria, we assigned scores to the studies ranging from 0 to 5. Studies reaching a score <3 were considered of poor quality and thus excluded from this review. Several studies, including systematic reviews, have already embraced this validated evaluation tool [84,85,86,87]. Furthermore, we used the risk-of-bias table proposed by *Cochrane* [88] to check the presence of selection, performance, detection, attrition and reporting biases in the selected clinical trials.

## 5. Conclusions

This review attempted to shed light on the anti-caries activity of EOs and their isolated constituents. Certainly, EOs extracted from a variety of aromatic plants worldwide can be considered promising sources of bioactive molecules effective against caries-related microorganisms, particularly *S. mutans*; however, most of the knowledge in the literature is based on *in vitro* studies and on a limited number of clinical trials. Overall, the studies have assessed the effects of EO and isolated compounds on microbial growth rather than virulence factors (e.g., bacterial EPS synthesis), which play a key role in the aetiopathogenesis of dental caries. Attention is also drawn to the fact that a number of studies do not provide any chemical or botanical characterization data, raising concern about the reproducibility and accuracy of their findings. Scientific journals should be more stringent in the adoption of criteria for the publication of studies with natural products, particularly EOs. Due to the gap between the *in vitro* biological properties identified in EOs and their clinical use for the prevention of dental caries, future researches should focus on translational approaches to advance the development of effective anti-caries products containing EO, given that most of them are considered as GRAS by the FDA.
